# Combinations of Radiotherapy with Vaccination and Immune Checkpoint Inhibition Differently Affect Primary and Abscopal Tumor Growth and the Tumor Microenvironment

**DOI:** 10.3390/cancers13040714

**Published:** 2021-02-09

**Authors:** Michael Rückert, Lisa Deloch, Benjamin Frey, Eberhard Schlücker, Rainer Fietkau, Udo S. Gaipl

**Affiliations:** 1 Department of Radiation Oncology, Friedrich-Alexander-Universität Erlangen-Nürnberg, Universitätsklinikum Erlangen, 91054 Erlangen, Germany; michael.rueckert@uk-erlangen.de (M.R.); lisa.deloch@uk-erlangen.de (L.D.); benjamin.frey@uk-erlangen.de (B.F.); rainer.fietkau@uk-erlangen.de (R.F.); 2 Institute of Process Machinery and Systems Engineering, Friedrich-Alexander-Universität Erlangen-Nürnberg, 91058 Erlangen, Germany; sl@ipat.uni-erlangen.de

**Keywords:** radiotherapy, vaccination, high hydrostatic pressure, immune checkpoint inhibition, abscopal effect, tumor-infiltrating immune cells, tumor microenvironment

## Abstract

**Simple Summary:**

Radiotherapy (RT) with higher single doses is capable of creating a beneficial immunological tumor microenvironment for additional immune therapies to trigger systemic anti-tumor immune responses. Here, we aimed to boost RT-induced immune responses with inactivated whole tumor cell vaccines generated with high hydrostatic pressure (HHP) and to break immunosuppression by inhibition of suppressive immune checkpoint molecules, such as PD-1. Our results highlight that HHP vaccines act systemically to retard tumor growth, but only of previously irradiated tumors. In contrast, abscopal anti-tumor immune responses, namely those to non-irradiated tumors, are triggered by a combination of RT of not too many fractions plus anti-PD-1, being characterized by the induction of distinct immune alterations in the primary and abscopal tumor, respectively. This emphasizes that future multimodal radioimmunotherapy approaches need to be carefully optimized regarding RT fractionation and the interaction of different immunotherapies with each other.

**Abstract:**

Radiotherapy (RT) is known to have immune-modulatory properties. We hypothesized that RT and inactivated whole tumor cell vaccines generated with high hydrostatic pressure (HHP) synergize to retard the tumor growth which can be additionally improved with anti-PD-1 treatment. In abscopal tumor models, we injected mice with B16-F10 melanoma or TS/A mammary tumors. To evaluate the efficiency of RT in combination with HHP vaccines, we locally irradiated only one tumor with 2 × 8 Gy or 3 × 8 Gy. HHP vaccines further retarded the growth of locally irradiated (2 × 8 Gy) tumors. However, HHP vaccination combined with RT failed to induce abscopal anti-tumor immune responses, namely those to non-irradiated tumors, and even partly abrogated those which were induced with RT plus anti-PD-1. In the latter group, the abscopal effects were accompanied by an elevated infiltration of CD8+ T cells, monocytes/macrophages, and dendritic cells. 3 × 8 Gy failed to induce abscopal effects in association with increased expression of immunosuppressive checkpoint molecules compared to 2 × 8 Gy. We conclude that HHP vaccines induce anti-tumor effects, but only if the tumor microenvironment was previously modulated by hypofractionated RT with not too many fractions, but failed to improve RT plus anti-PD-induced abscopal responses that are characterized by distinct immune alterations.

## 1. Introduction

The treatment of a tumor with ionizing radiation is known to induce immunogenic cell death in tumor cells under certain conditions, which is characterized by the release of damage-associated molecular patterns (DAMPs), tumor antigens, pro-inflammatory cytokines, and others, which turns them into an in situ cancer vaccine. Dendritic cells (DCs) take up the tumor antigen, transport it to the draining lymph node, and present it there to T cells, which are subsequently released into the periphery [1,2]. In an ideal situation for a patient with metastasized disease, local radiotherapy (RT) of the primary tumor or a metastasis thereby triggers a systemic immune response also directed against further lesions in the body including distant, non-irradiated tumors. The resulting shrinkage of the tumor masses outside the irradiation field is the so-called abscopal effect and was first theoretically described and termed by Mole et al. in 1953 [3]. In contrast to the “non-targeted” effects of ionizing radiation, namely bystander (about 5 mm away from the irradiated part) or distant effects (about 5 cm away from the irradiated part), abscopal effects occur on a more distant site of the irradiated area [4]. In times of combination of RT with immunotherapies (IT), abscopal effects are related to anti-tumor effects of RT that are observed outside of the radiation field. They are mediated by the immune system [5]. In a proof of principle study with non-small cell lung cancer and breast cancer, it was shown that local RT plus GM-CSF can induce such abscopal immune responses in patients [6]. However, the abscopal effect was rarely seen in the clinic, with only about one reported case per year in the literature between 1969 and 2014 [7]. In consequence of the ambivalent effects of RT with simultaneous immune stimulation and modulation of the tumor microenvironment (TME), but also suppression of emerging and pre-existing anti-tumor immune responses [8], RT alone is often not enough to fully eradicate tumors and to induce abscopal effects. Therefore, ITs have great potential to complement RT by further boosting RT-induced immune responses and/or counteracting tumor-intrinsic as well as RT-mediated immunosuppression [9]. This beneficial liaison of combinations of RT and ITs is reflected by the continuously increasing reports of abscopal effects since the approval of the immune checkpoint inhibitor ipilimumab in 2011 [10] and ITs in general [11].

Immune checkpoint inhibitors (ICIs) targeting the immunosuppression of cytotoxic T lymphocytes (CTLs) in the priming (anti-CTLA-4) and effector phase (anti-PD-1, anti-PD-L1/2) have become the most promising and effective ITs in the last decade. Even though the number of long-term survivors increased, a large proportion of patients remains refractory to the treatment, which calls for further improvements, such as combined therapies [12,13].

The inactivation of tumor cells with high hydrostatic pressure (HHP) is a suitable method to generate whole tumor cell vaccines potentially being applied as autologous vaccination. Non-physiological HHP above 100 MPa (1000 bar) has irreversible effects and eventually leads to cell death [14]. Without changing the primary structure, HHP denatures proteins only by affecting their tertiary and quaternary structure and thereby inactivates enzymes [15]. Although human cells tend to be more resistant to HHP than murine cells, pressure of at least 200 MPa effectively and reproducibly kills tumor cells of both species [16]. HHP treated cells predominantly die an immunogenic cell death characterized by DNA degradation at later time points, phosphatidylserine exposure, loss of membrane integrity, and the release of DAMPs, such as HMGB1, HSP70, HSP90, and ATP [16,17,18]. Therefore, HHP was included in the list of immunogenic cell death inducers [19]. In addition to the adjuvanticity HHP treated tumor cells are given by these DAMPs, Urbanova et al. reported that HHP preserves tumor antigens if the pressure is not too high, that HHP treated tumor cells are phagocytosed by DCs, and that these DCs subsequently stimulate CD8+ T cells [20]. They conclude that 200 MPa is the optimal pressure as a compromise of antigen degradation and immunogenicity. Currently, HHP treated tumor cells are tested as therapeutic cancer vaccines in two phase I/II clinical trials for ovarian and lung cancer (NCT03657966, NCT02470468) and one phase III trial for prostate cancer (NCT02111577). In these trials, monocyte-derived DCs from patients are loaded with HHP treated allogeneic tumor cell lines (DCVAC) followed by the injection of these pulsed DCs back into the patient.

Recently, we have shown that, upon peritumoral injection, HHP vaccines synergize with RT to retard the tumor growth by generating a favorable TME [21]. We now investigated the induction of systemic anti-tumor immune responses by RT plus HHP vaccination with distant injection and anti-PD-1 checkpoint inhibition and how these treatments differently affect the TME of primary and abscopal tumors.

We found that HHP vaccines work systemically, but only on previously irradiated tumors and that abscopal responses to RT plus anti-PD1 are even weakened by HHP vaccines and if the number of fractions of RT was too high, which was associated with upregulation of suppressive immune checkpoint ligands.

## 2. Materials and Methods

### 2.1. Cell Lines and Cell Culture

Commercially available (ATCC, Manassas, VA, USA) murine B16-F10 melanoma cells derived from C57Bl/6 mice and TS/A mammary adenocarcinoma derived from BALB/c mice, which were kindly provided by Prof. Lollini (Bologna, Italy), were cultured in RPMI 1640 supplemented with 10% fetal bovine serum without addition of any antibiotics under standard conditions in an incubator with 37 °C, 95% humidity, 5% CO_2_. Authenticity was assured by cell banking, morphology, and usage of low-passage cells cultivated for a maximum of 10 passages. The cell lines were routinely tested for mycoplasma contamination by PCR.

### 2.2. High Hydrostatic Pressure (HHP) Treatment

For the preparation of the whole tumor cell vaccine, adherent tumor cells were detached with Accutase (Merck, Darmstadt, Germany) to obtain single cell suspensions. The cells were washed with PBS (Sigma-Aldrich, St. Louis, MO, USA) and resuspended in Ringer solution (Fresenius Kabi, Bad Homburg vor der Höhe, Germany) at a concentration of 25 × 10^6^ cells/mL. Cryogenic vials (Greiner, Kremsmünster, Austria) containing the tumor cells were pressurized in the computer-controlled HHP treatment system which was provided by the “*Lehrstuhl für Prozessmaschinen und Anlagentechnik*” (iPAT, Friedrich-Alexander-Universität Erlangen-Nürnberg). The high hydrostatic pressure treatment of the vials with tumor cells was conducted with a pressure build-up velocity of 50 MPa/s and a final pressure of 200 MPa for 300 s. Previously, it was shown that DAMPs particularly accumulate in the supernatant of cells treated with 200 MPa after 24 h [20]. Therefore, the cells were incubated for 24 h in the incubator without changing the medium to avoid the loss of any released DAMPs before using them for in vitro experiments or as a vaccine in vivo.

### 2.3. Animal Experiments

For each experiment of the abscopal tumor models, B16-F10 tumor cells were freshly thawed, cultivated for three passages, and then 0.2 × 10^6^ cells were injected subcutaneously into the right (day 7) and left (day 3) flank of syngeneic 6–8 week old male C57Bl/6 mice (Janvier, Le Genest-Saint-Isle, France). Only the first injected primary right flank tumor was irradiated with 8 Gy after seven days (d0) as described in [22]. On day 3 (2 × 8 Gy experiments) or on days 1 and 3 (3 × 8 Gy experiments), the mice received another dose of 8 Gy. Beginning with the first irradiation on d0, some mice were intraperitoneally injected with 200 µg anti-PD-1 antibody (αPD-1, RMP1-14) (Bio X Cell, Lebanon, NH, USA) every three to four days for a total of four injections. Additionally, the HHP vaccine (5 × 10^6^ cells) was injected twice subcutaneously into the neck on days 2 and 8. Tumor and blood samples were collected from some animals on day 8 for cytokine analyses and on day 10 for immune phenotyping. Tumor growth experiments with TS/A tumors were conducted accordingly, except tumor inoculation which was done on day 12 (primary) and day 10 (abscopal) with 0.1 × 10^6^ cells in BALB/c mice. During and after the treatment in all in vivo experiments, the tumor growth of the mice was examined. The tumor volume was calculated with the formula (length × width2)/2. Once one of the tumors exceeded a volume of 1500 mm^3^, the mice were sacrificed due to ethical reasons.

### 2.4. Immune Phenotyping of Blood and Tumor Infiltrating Immune Cells

Leukocyte subtypes infiltrating into tumors and in the peripheral blood were analyzed on day 10 of the treatment period. The tumors were weighed before dissociation with the tumor dissociation kit (Miltenyi, Bergisch Gladbach, Germany). Immune cells were isolated from up to 0.3 g of tumor with CD45 (TIL) MicroBeads (Miltenyi, Bergisch Gladbach, Germany) by MACS separation. The flow through, predominantly containing tumor cells, was collected and the cells were stored in TriFast (VWR, Radnor, PA, USA) for further analyses. For the immune phenotyping of blood erythrocytes of 100 µL, whole blood were lysed using a TQ-Prep Workstation (Beckman Coulter, Brea, CA, USA). Both, tumor and blood samples were resuspended in 50 µL Fc-block solution (eBioscience, San Diego, CA, USA) and incubated for 10 min at room temperature. The cells were stained for 30 min at 4 °C with three different antibody panels (Tables S1–S3). The gating strategies for the immune phenotyping panels are displayed in Figures S1–S3. For the intracellular staining of FoxP3 in panel 3 (Figure S3), the cells were fixed and permeabilized using the FoxP3 staining buffer set (Miltenyi, Bergisch Gladbach, Germany). The antibody panels are provided in the Supplementary Materials. The cells were then washed two times and analyzed with a CytoFLEX S flow cytometer (Beckman Coulter, Brea, CA, USA). The data were analyzed with the Kaluza analysis software (Beckman Coulter, Brea, CA, USA). The concentration of tumor infiltrating immune cells per gram of tumor was calculated.

### 2.5. In Vitro Irradiation Experiments

For the in vitro investigation of the influence of different fractionations on tumor cells, the cells were seeded 16 h before the experiment and irradiated with X-rays using an X-ray tube in a lead shielding chamber. The 2 × 8 Gy treatment group was irradiated on d0 and d3, the 3 × 8 Gy group on d0, d1, and d3. The single dose treatment groups (1 × 8 Gy, 1 × 20 Gy) were irradiated on d3 only and one group was left untreated. On d4, 24 h after the last irradiation, the cells of one flask including the dead cells were detached with Accutase and used for subsequent flow cytometric analysis. Cell-free supernatant was collected from the other flask before detaching the cells with Accutase as well. Those cells were resuspended in TriFast for qPCR analysis.

### 2.6. Cell Death Analysis

Apoptotic and necrotic cell death of tumor cells was determined via flow cytometry. The staining was performed with FITC-labeled AnnexinA5 (AxV) and propidium iodide (PI) (Sigma-Aldrich, St. Louis, MO, USA). 0.2 × 10^6^ tumor cells were resuspended in cell death staining solution (1 µL PI and 0.75 µL AxV-FITC per 1 mL Ringer solution). After incubation for 30 min at 4 °C, the cells were analyzed with a CytoFLEX S flow cytometer. AxV^−^, PI^−^ cells were defined as viable, AxV^+^, PI^−^ as apoptotic, and AxV^+^, PI^+^ as necrotic.

### 2.7. Cell Cycle Analysis

For cell cycle analysis, 900 µL ethanol (70%, −20 °C) was added to 0.4 × 10^6^ tumor cells resuspended in 100 µL PBS. After incubation of at least 20 min at −20 °C, the cells were resuspended in 500 µL PBS and the cells were permeabilized by adding 500 µL permeabilization buffer for 5 min. After centrifugation, the cell pellet was resuspended in 500 µL DNA staining solution containing PI (5 µg/mL) and RNase (200 µg/mL) and was then incubated for 30 min at room temperature in the dark. At least 0.2 × 10^6^ cells were analyzed with a CytoFLEX S flow cytometer.

### 2.8. Immune Checkpoint Ligands

The surface expression of immune checkpoint ligands on tumor cells was analyzed by flow cytometry. For this, 0.2 × 10^6^ cells were first resuspended in Fc-block solution for 10 min at room temperature and then in antibody mix of “checkpoint panel 1” (Table S4), “checkpoint panel 2” (Table S5), or the respective autofluorescence control (AF ctrl) mix only containing the viable/dead stain, and incubated for 30 min at 4 °C. The cells were then washed two times and analyzed with a CytoFLEX S flow cytometer. As irradiated tumor cells tend to have a higher autofluorescence (AF) than untreated cells, the median fluorescence intensity (MFI) of each immune checkpoint ligand in the stained sample was corrected for this background with the AF ctrl. Thus, the ΔMFI was calculated by subtracting the MFI of the AF ctrl from the MFI of the stained sample.

### 2.9. Cytokine Analyses from Tumor Lysates and Serum

To prepare tumor lysates for protein analyses, the tumors were excised and weighed. Per 0.1 g of tumor, a volume of 2.5 mL tumor lysis buffer containing 1X Lysis Buffer (RayBiotech, Peachtree Corners, GA, USA), 1 mM PMSF protease inhibitor and 1X HALT protease inhibitor cocktail (both Thermo Fisher, Waltham, MA, USA), 1:30 Antifoam Y-30, 1 mM sodium fluoride, 1 mM β-glycerophosphate, and 0.2 mM sodium orthovanadate (all from Sigma-Aldrich, St. Louis, MO, USA) was added in a gentleMACS M tube and the tumors were dissociated with a gentleMACS dissociator. The protein concentration was determined with the Pierce BCA Protein Assay Kit (Thermo Fisher, Waltham, MA, USA).

To examine cytokines in cell culture supernatants and tumor lysates, the following ELISAs were performed according to the manufacturer’s recommendations unless stated otherwise: HSP70 (supernatants, undiluted), HMGB1 (supernatants, undiluted), CXCL1 (supernatants, undiluted), IFN-β (supernatants, undiluted; tumor lysate, 100 µg total protein). The OD at 450 nm and 540 nm as reference wavelength was recorded with an Epoch Microplate Spectrophotometer (BioTek, Winooski, VT, USA). For the analysis of IFN-γ and TNF-α in tumor lysates and in serum, the V-PLEX Proinflammatory Panel 1 Mouse Kit (MSD, Rockville, MD, USA) was used. The assay was conducted according to the manufacturer’s instructions and the plates were read on a MESO QuickPlex SQ 120 (MSD, Rockville, MD, USA).

### 2.10. Quantitative Real-Time PCR (qPCR)

Pre-designed Prime PCR Primers from Bio-Rad (Hercules, CA, USA) were used for all qPCR reactions (Supplementary Table S6). The qPCR analysis was carried out with the DyNAmo ColorFlash SYBR Green qPCR Kit (Thermo Fisher, Waltham, MA, USA). The qPCR results were analyzed with the Bio-Rad CFX Manager software and the gene expression was calculated as normalized gene expression to the three housekeeping genes *Hprt*, *Tbp*, and *Rps18*.

### 2.11. Statistical Analyses

Statistical analysis was performed with the GraphPad Prism software (GraphPad Software, San Diego, CA, USA). Unless indicated otherwise, a Kruskal–Wallis test was calculated to compare all treatment groups against the untreated control with Dunn’s correction for multiple testing and a Mann–Whitney U test to compare two treatment groups. For the survival a log-rank test with Holm–Sidak correction for multiple testing was applied. Significances were indicated as follows: * *p* < 0.05, ** *p* < 0.01, *** *p* < 0.001.

## 3. Results

### 3.1. RT-Mediated Local Tumor Control of the Primary Tumor Can Be Improved with Immunotherapies but Abscopal Responses Are Only Induced Together with Anti-PD-1

**Figure 1:**
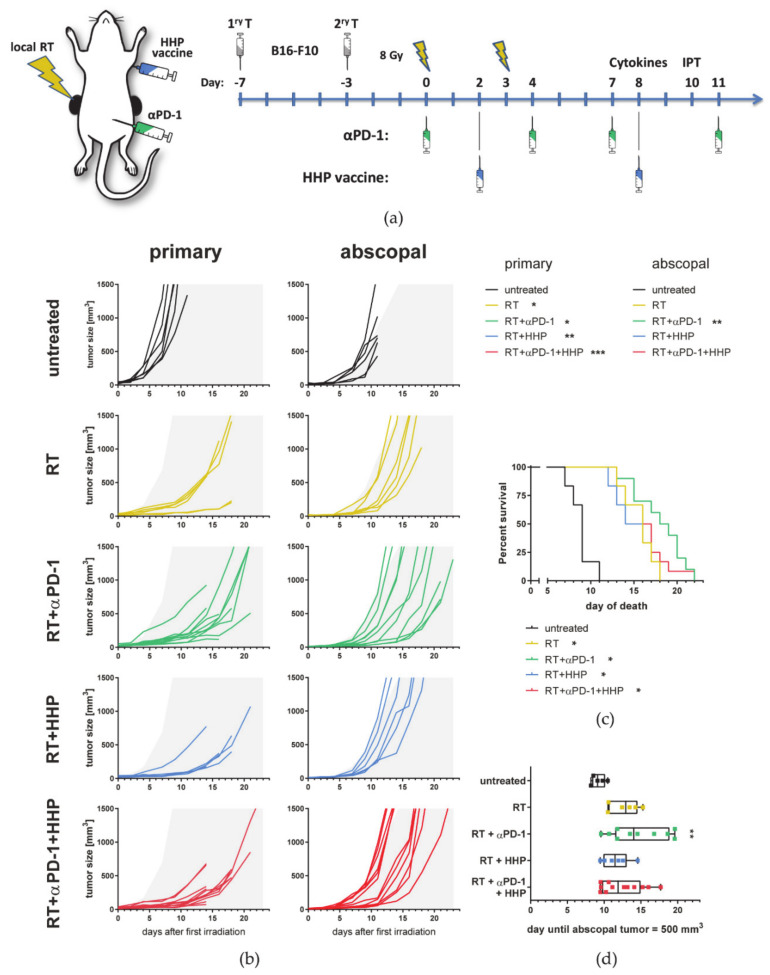
High hydrostatic pressure (HHP) vaccines act systemically but only on previously irradiated tumors and fail to improve RT + anti-PD-1 induced abscopal responses. (**a**) C57Bl/6 mice were subcutaneously injected with 0.2 × 10^6^ B16-F10 tumor cells into the right flank. Four days afterwards, a second tumor was injected on the left flank which later served as the non-irradiated abscopal tumor. The mice received one of the following treatments or combinations thereof. Only the first injected primary tumor was irradiated with 2 × 8 Gy on d0 seven days after injection and on d3. Beginning with the first irradiation on d0, the mice were intraperitoneally injected with 200 µg anti-PD-1 antibody (αPD-1) every three to four days for a total of four injections. Additionally, the HHP vaccine (5 × 10^6^ cells) was injected twice subcutaneously into the neck on days 2 and 8. Tumor and blood samples were collected from some animals on day 8 for cytokine analyses or on day 10 for immune phenotyping, respectively. (**b**) Individual tumor growth curves are depicted. For a better comparability of the treatment groups, gray areas indicate retarded tumor growth beyond the mean of primary and abscopal tumors of the control group, respectively. A Kruskal–Wallis test with Dunn’s correction for multiple testing was calculated to compare the areas under the individual growth curves of the treatments with untreated controls. (**c**) For the survival a log-rank (Mantel–Cox) test was calculated with Holm–Sidak correction for multiple testing to compare the treatments with the control group. (**d**) The time until the abscopal tumors reached a volume of 500 mm^3^ is depicted as box and whiskers plot and a Kruskal–Wallis test with Dunn’s correction for multiple testing was calculated to compare treatments with untreated controls. * *p* < 0.05, ** *p* < 0.01, *** *p* < 0.001; *n* = 6–12 animals per group from two independent experiments.

We first aimed to investigate if combinations of RT plus HHP vaccine, which is injected distantly from both tumors, are capable of inducing anti-tumor immune responses in the locally irradiated and in the non-irradiated abscopal tumor. Increasing evidence suggests that hypofractionated treatment schedules are superior to normfractionation in eliciting the most favorable immune response by fostering ICD induction and immune cell infiltration [10,23,24,25], although a certain threshold in the dose per fraction should not be exceeded [26,27]. Therefore, we have chosen to irradiate tumors with 2 × 8 Gy. Based on knowledge about the high percentage of PD-1+ T cells after the RT plus peritumoral HHP vaccination [21], we also included anti-PD-1 immune checkpoint blockade in the treatment schedule (Figure 1a). Therefore, C57Bl/6 mice were injected with one tumor on each flank at a timely distance of 4 days, and only the first injected primary tumor was locally irradiated. Anti-PD-1 mAbs were administered concurrently with the RT and the HPP vaccine was applied twice by subcutaneous injection in the neck.

RT and RT plus anti-PD-1 both significantly improved the local tumor control of the irradiated tumor compared to the untreated control (Figure 1b). Although injected distantly from the primary irradiated tumor, the HHP vaccine with or without additional anti-PD-1 treatment was also capable to further retard the tumor growth of the irradiated tumor.

In contrast to RT alone, the addition of anti-PD-1 significantly retarded the tumor growth of the non-irradiated abscopal tumor. HHP vaccination had no prominent effect on the abscopal tumor growth and even partly abrogated RT plus anti-PD-1-mediated abscopal responses. Even though RT significantly prolonged the survival of the mice in all settings due to inducing good local tumor control, the animals with the longest survival were in the RT plus anti-PD-1 groups (Figure 1c). As the response to RT plus anti-PD-1 was heterogeneous in abscopal tumors as previously observed in other models [23], we additionally calculated the time until the abscopal tumors reached a volume of 500 mm^3^ [28]. Although tumor growth was slightly retarded in most irradiated animals, only RT plus anti-PD-1 significantly delayed the abscopal tumor growth compared to the untreated control (Figure 1d).

**Figure 2:**
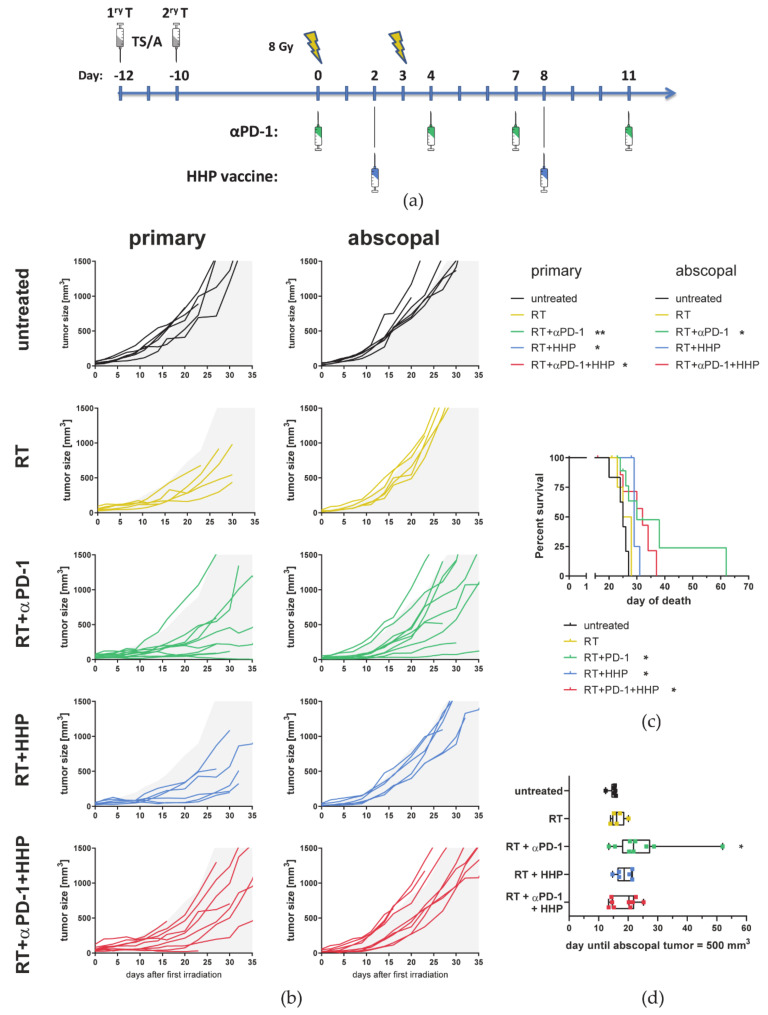
High hydrostatic pressure (HHP) vaccines act systemically but only on previously irradiated tumors and fail to improve RT + anti-PD-1 induced abscopal responses also in the TS/A tumor model. (**a**) BALB/c mice were subcutaneously injected with 0.2 × 10^6^ TS/A tumor cells into the right flank. Two days afterwards, a second tumor was injected on the left flank which later served as the non-irradiated abscopal tumor. The mice received one of the following treatments or combinations thereof. Only the first injected primary tumor was irradiated with 2 × 8 Gy on d0 (12 days after injection) and on d3. Beginning with the first irradiation on d0, the mice were intraperitoneally injected with 200 µg anti-PD-1 antibody (αPD-1) every three to four days for a total of four injections. Additionally, the HHP vaccine (5 × 10^6^ cells) was injected twice subcutaneously into the neck on days 2 and 8. (**b**) Individual tumor growth curves are depicted. For a better comparability of the treatment groups, gray areas indicate retarded tumor growth beyond the mean of primary and abscopal tumors of the control group, respectively. A Kruskal–Wallis test with Dunn’s correction for multiple testing was calculated to compare the areas under the individual growth curves of the treatments with untreated controls. (**c**) For the survival a log-rank (Mantel–Cox) test was calculated with Holm–Sidak correction for multiple testing to compare the treatments with the control group. (**d**) The time until the abscopal tumors reached a volume of 500 mm^3^ is depicted as box and whiskers plot and a Kruskal–Wallis test with Dunn’s correction for multiple testing was calculated to compare treatments with untreated controls. * *p* < 0.05, ** *p* < 0.01; *n* = 5–10 animals per group from two independent experiments.

A similar benefit of HHP vaccination on previously irradiated primary tumors, but not on abscopal ones, and of beneficial effects of RT plus anti-PD-1 treatment on abscopal tumor growth, was also observed in the TS/A mammary carcinoma model (Figure 2).

### 3.2. Immune Cell Profiles Differ between Primary and Abscopal Tumors and between the Treatment Groups

As primary and abscopal tumors responded differently to the immunotherapies, we hypothesized that the immune cell composition will differ between primary and abscopal tumors and between the treatment groups. Therefore, we performed multi-color flow cytometry on day 10, one week after the last fraction to identify tumor infiltrating leukocyte subtypes. The different cell types were identified according to the gating strategies shown in Figures S1–S3.

**Figure 3:**
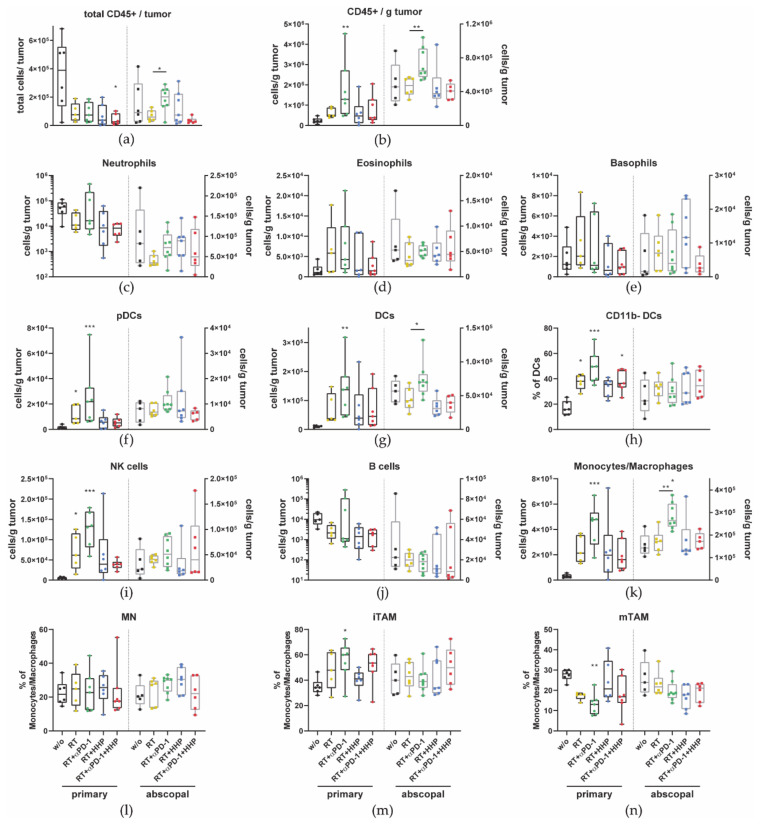
Combinations of radiotherapy (RT) with immunotherapies differently affect tumor-infiltrating immune cell types in primary and abscopal tumors. On day 10 after first irradiation, mice were sacrificed and immune cells infiltrating into the tumors were analyzed by multi-color flow cytometry. The total amount of all immune cells (CD45+) (**a**) and the concentration per gram of tumor (**b**) in primary and abscopal tumors are displayed. Immune cell subtypes were identified as follows: neutrophils CD11b+, Ly6G+ (**c**); eosinophils CD11b+ Siglec-F+ (**d**); basophils FcεR1α+, CD49b+ (**e**); plasmacytoid dendritic cells (pDCs) CD11b-, Ly6G-, PDCA-1+, Ly6C+ (**f**); dendritic cells (DCs) MHC-II+, CD11c+ (**g**), which were further subdivided in CD11b- cDC1s (**h**); natural killer (NK) cells CD49b+, CD3- (**i**); B cells CD19+ (**j**); monocytes/macrophages CD11b+, Ly-6C+ (**k**), which were further subdivided in Ly6C+, MHC-II- monocytes (MN, (**l**)), Ly6C+, MHC-II+ immature tumor associated macrophages (iTAM, (**m**)); and Ly6C- mature TAM (mTAM, (**n**)). Data are presented as box plots with whiskers from minimum to maximum values. A Kruskal­–Wallis test with Dunn’s correction for multiple testing was used to compare the primary and abscopal tumors of all treatment groups with the respective untreated control (w/o) tumors. Additionally, a Mann–Whitney U test was calculated to compare RT + anti-PD-1 and RT groups; * *p* < 0.05, ** *p* < 0.01, *** *p* < 0.001; *n* = 5–8 animals per group from two independent experiments.

Although the total amount of immune cells (CD45+) in the primary tumors after any form of treatment was less than that in untreated controls (Figure 3a), the concentration of CD45+ cells per gram of tumor mass was significantly increased in primary tumors of the RT plus anti-PD-1 group (Figure 3b). Additionally, in this treatment group, which also showed the most pronounced abscopal responses, the total amount of immune cells and the concentration in the abscopal tumor were significantly higher than after RT alone.

Higher concentrations of pDCs, DCs, NK cells, and monocytes/macrophages were detected in primary tumors that had been treated with RT and they even further increased after the addition of anti-PD-1 (Figure 3f,g,i,k). By further subdividing the DC and monocytes/macrophages compartment, we found that only in primary tumors the percentage of the CD11b- DC (cDC1) subtype was significantly higher after irradiation and even higher with addition of anti-PD-1 (Figure 3h). No changes were observed for the percentage of monocytes (MN) among all treatment groups (Figure 3l). In primary tumors treated with RT plus anti-PD-1, the fraction of immature tumor-associated macrophages (iTAMs) significantly increased, accompanied with a decrease in mature TAMs (mTAMs) (Figure 3m,n).

No significant changes were observed for all granulocyte subtypes (neutrophils, eosinophils, and basophils) and B cells (Figure 3c–e,j). However, in all irradiated primary tumors, the concentrations of neutrophils and B cells tended to be decreased.

**Figure 4:**
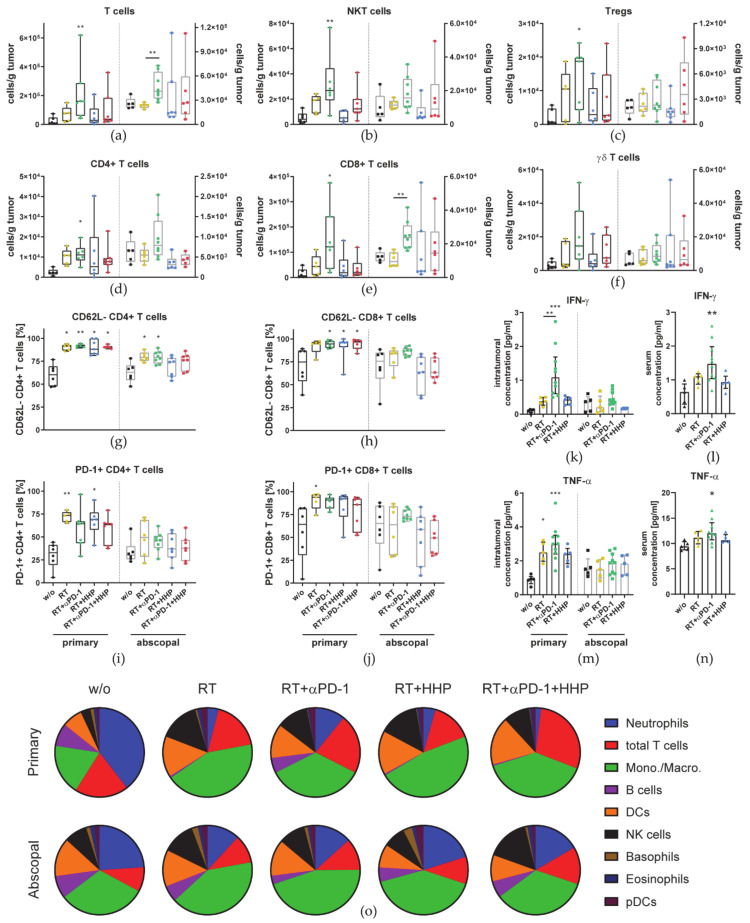
2 × 8 Gy plus anti-PD-1 increases the concentration of tumor-infiltrating T cell subpopulations in primary and abscopal tumors. On day 10 after first irradiation, mice were sacrificed and immune cells infiltrating into the tumors were analyzed by multi-color flow cytometry. (**a**–**f**) The concentration of immune cells per gram of primary and abscopal tumor is displayed. Immune cell subtypes were identified as follows: T cells CD3+, CD49b- (**a**); NKT cells CD3+, CD49b+ (**b**); regulatory T cells (Tregs) CD3+, CD4+, CD25+ FoxP3+ (**c**); CD4+ T cells (**d**); CD8+ T cells (**e**); γδ T cells CD3+, γδTCR+ (**f**). CD4+ (**g**,**i**) and CD8+ (**h**,**j**) T cells were analyzed for their CD62L (**g**,**h**) and PD-1 (**i**,**j**) expression. Data are presented as box plots with whiskers from minimum to maximum values. On day 8 after the first irradiation of the 2 × 8 Gy irradiation, mice were sacrificed for the analysis of cytokines in tumors (**k**,**m**) and the serum (**l**,**n**) with multiplex-ELISA. Data for IFN-γ (**k**,**l**) and TNF-α (**m**,**n**) are presented as bar graphs with individual values and interquartile range. A Kruskal–Wallis test with Dunn’s correction for multiple testing was used to compare all treatment groups with the respective untreated control (w/o). Additionally, a Mann–Whitney U test was calculated to compare RT + anti-PD-1 and RT groups. (**o**) The compositions of major immune cell types in relation to each other of primary and abscopal tumors are displayed as pie charts. Each cell type is represented as the mean of all tumors of each group.; * *p* < 0.05, ** *p* < 0.01, *** *p* < 0.001, *n* = 5–8 animals per group from two independent experiments.

Further, we also observed an increased concentration of T cells in primary and abscopal tumors of the RT plus anti-PD-1 group (Figure 4a). Additionally, T cells were further subdivided and analyzed for their PD-1 and CD62L expression. The concentration of NKT cells, Tregs, and both, CD4+ and CD8+ T cells, in primary tumors of the RT plus anti-PD-1 treatment group was significantly higher than that in untreated control tumors (Figure 4b–e). Although not significant, the same trend was observed for γδ T cells (Figure 4f).

Only CD8+ T cells, monocytes/macrophages, and DCs were found in significantly higher concentrations in abscopal tumors of the RT plus anti-PD-1 treatment group compared to RT alone (Figure 3g,k and Figure 4e). RT increased the proportion of PD-1+ and CD62L- cells among CD4+ and CD8+ T cells in primary tumors, albeit only to a significant degree in some treatment combinations including RT (Figure 4g–j). In abscopal tumors, only the percentage of CD62L- CD4+ T cells was significantly increased in the RT and RT plus anti-PD-1 groups (Figure 4g).

Cytokines represent another important factor in the tumor microenvironment. To investigate how the combination of each individual immunotherapy with RT modulates the cytokine profile in the tumor and how these influence the infiltration of immune cells, we generated tumor lysates for multiplex-ELISA. Additionally, the concentration of those cytokines was analyzed in the serum.

The concentration of the most prominent cytokines, IFN-γ and TNF-α, was found to be significantly higher in primary tumors of the RT plus anti-PD-1 treatment group than in untreated control tumors (Figure 4k,m). No changes were detected in abscopal tumors. In serum, the level of IFN-γ and TNF-α in the RT plus anti-PD-1 group was significantly elevated as well (Figure 4l,n).

To get a better overview on the relative abundance of the main immune cell types identified before (Figure 3 and Figure 4) in relation to each other and how this changes upon treatment, we visualized the composition of primary and abscopal tumors of each group (Figure 4o).

Neutrophils, T cells, and monocytes/macrophages together made up more than 75% of all identified major immune cell types in the untreated primary tumor. Among all treatment groups, this composition in primary tumors was predominantly changed by RT characterized with less neutrophils, B cells, and more monocytes/macrophages, DCs and NK cells. However, the composition in abscopal tumors was not drastically changed in the treatment groups.

**Figure 5:**
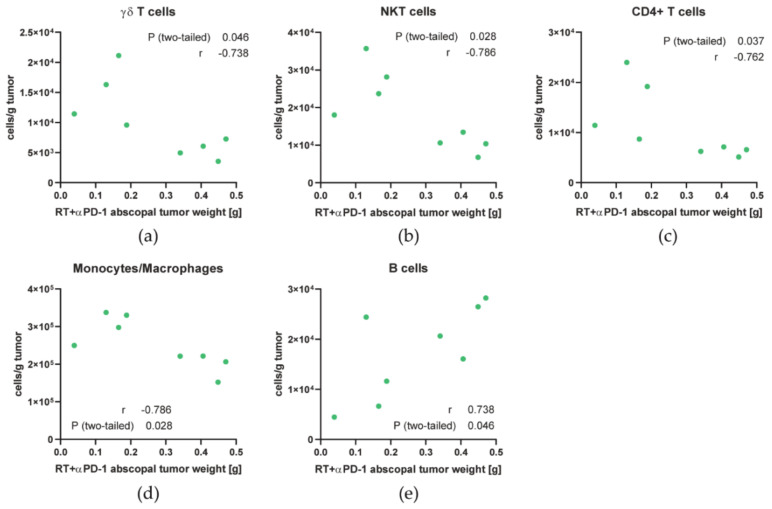
The abscopal tumor growth in the 2 × 8 Gy + anti-PD-1 group correlates with the infiltration of multiple immune cells. (**a**–**e**) Spearman correlation was calculated for the abscopal tumor weight of the RT + anti-PD-1 group with concentration of γδ T cells (**a**), NKT cells (**b**), CD4+ T cells (**c**), monocytes/macrophages (**d**), and B cells (**e**).

As the response of abscopal tumors to RT plus anti-PD-1 was quite heterogeneous, we analyzed which cell types might contribute to abscopal responses by correlating the immune cell concentrations with the weight of the abscopal tumor. Additionally to the generally increased infiltration of monocytes/macrophages and T cells in those tumors, higher concentrations of monocytes/macrophages and the γδ, NKT and CD4+ T cell subsets correlated with good abscopal responses (lower tumor weight) in those tumors (Figure 5a–d). In contrast, B cell numbers positively correlated with abscopal tumor growth (Figure 5e).

**Figure 6:**
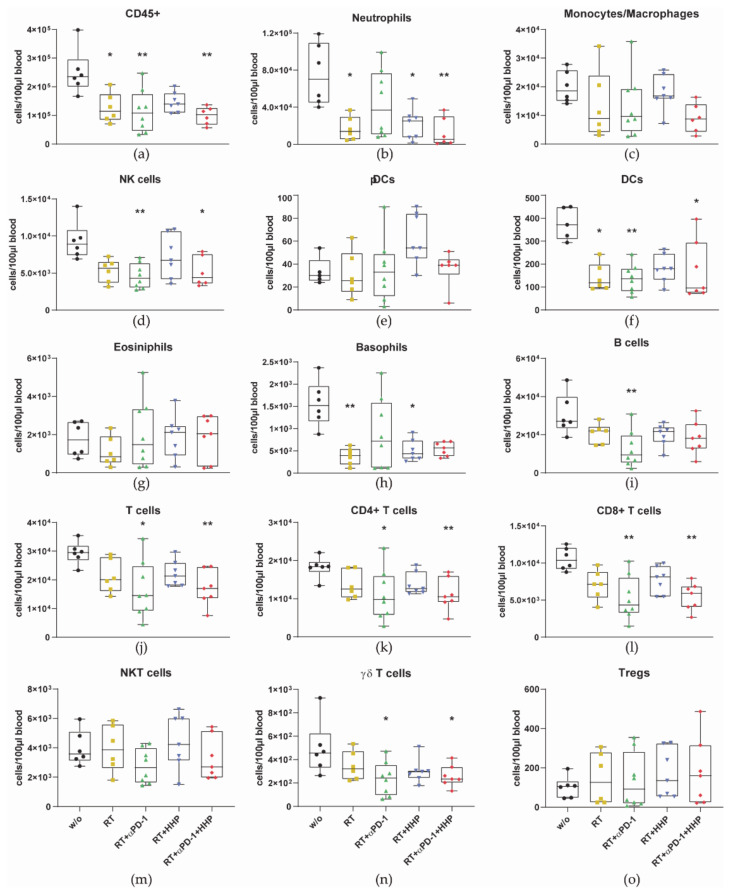
Immune cell numbers drop in the peripheral blood after irradiation with 2 × 8 Gy. On day 10 after first irradiation, mice were sacrificed and immune cells in the blood were analyzed by multi-color flow cytometry. (**a**–**o**) The concentration of immune cells per 100 µL blood is displayed. Immune cell subtypes were identified as follows: all immune cells CD45+ (**a**); neutrophils CD11b+, Ly6G+ (**b**); monocytes/macrophages CD11b+, Ly-6C+ (**c**); natural killer (NK) cells CD49b+, CD3- (**d**); plasmacytoid dendritic cells (pDCs) CD11b-, Ly6G-, PDCA-1+, Ly6C+ (**e**); dendritic cells (DCs) MHC-II+, CD11c+ (**f**); eosinophils CD11b+ Siglec-F+ (**g**); basophils FcεR1α+, CD49b+ (**h**); B cells CD19+ (**i**); total T cells CD3+, CD49b- (**j**); CD4+ (**k**); and CD8+ (**l**) T cells; NKT cells CD3+, CD49b+ (**m**); γδ T cells CD3+, γδTCR+ (**n**); regulatory T cells (Tregs) CD3+, CD4+, CD25+ FoxP3+ (**o**). Data are presented as box plots with whiskers from minimum to maximum values. A Kruskal–Wallis test with Dunn’s correction for multiple testing was used to compare all treatment groups with the untreated control (w/o); * *p* < 0.05, ** *p* < 0.01; *n* = 6–8 animals per group from two independent experiments.

Additionally, to the lower immune cell numbers in irradiated tumors, immune cells in general (CD45+) and most of the cell types decreased in the peripheral blood after RT as well (Figure 6).

### 3.3. Combinations of RT with Immunotherapies Increase the Expression of Immune Checkpoint Ligands in Primary Tumors

Next to the infiltration of immune cells into the tumors, the tumor microenvironment they migrate into is also of relevance for the induction and the effector phase of the anti-tumor immune response. Therefore, we analyzed the mRNA expression of immune checkpoint ligands in the tumor microenvironment. The samples for qPCR were taken from the flow through of the CD45+ MACS separation for the immune phenotyping and thus contain all cells of the tumor with the exception of immune cells.

**Figure 7:**
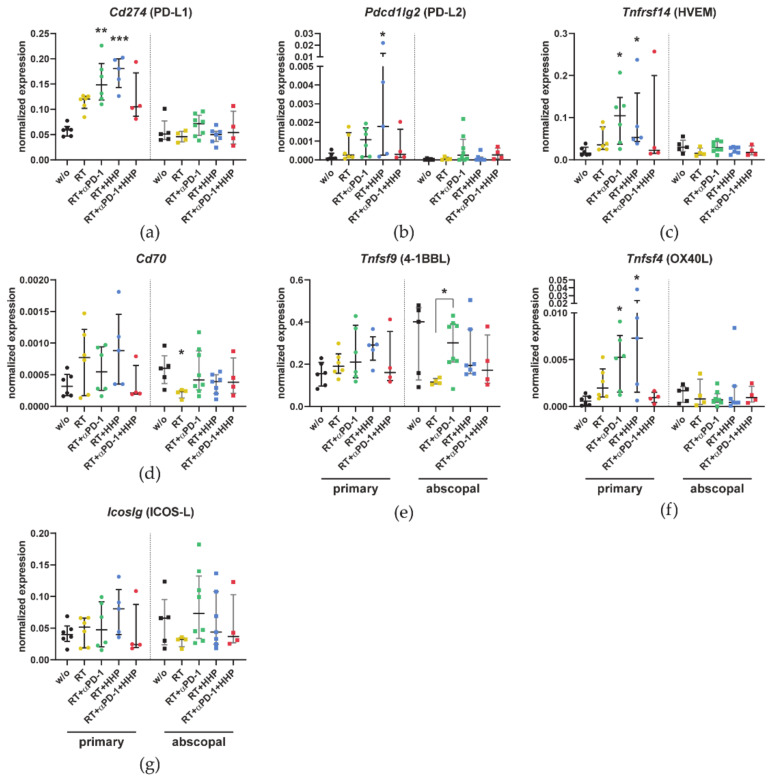
Combinations of RT with immunotherapies affect the expression of immune checkpoint ligands in primary tumors. On day 10, after first irradiation of the 2 × 8 Gy irradiation mice were sacrificed and the primary and abscopal tumors were dissociated. The flow through of the CD45-MACS containing all non-immune cells was collected for qPCR analysis. (**a**–**g**) The expression of *Cd274* (**a**), *Pdcd1lg2* (**b**), *Tnfrsf14* (**c**), *Cd70* (**d**), *Tnfsf9* (**e**), *Tnfsf4* (**f**), and *Icoslg* (**g**) is displayed as normalized expression to the three housekeeping genes *Hprt*, *Tbp*, and *Rps18*. Data are presented as median ± interquartile range. A Kruskal–Wallis test with Dunn’s correction for multiple testing was used to compare all treatment groups with the untreated control (w/o). Additionally, a Mann–Whitney U test was calculated to compare RT + anti-PD-1 and RT groups; * *p* < 0.05, ** *p* < 0.01, *** *p* < 0.001; *n* = 4–8 animals per group from two independent experiments.

The expression of *Cd274* (PD-L1) in primary tumors was about two-fold higher in the RT and triple combination groups and up to three-fold higher in the RT plus anti-PD-1 or plus HHP vaccine groups compared to untreated controls (Figure 7a). Similar trends were observed for the other two inhibitory immune checkpoint ligands *Pdcd1lg2* (PD-L2) and *Tnfrsf14* (HVEM) (Figure 7b,c), albeit the *Pdcd1lg2* (PD-L2) expression was only significantly elevated after RT plus HHP vaccination and the overall expression of this ligand was quite low. In contrast, the changes in the expression of the stimulatory immune checkpoint ligands *Cd70*, 4-1BBL, *Tnfsf9* (OX40L), and *Tnfsf4* (ICOS-L) were less pronounced (Figure 7d–g). Only the mRNA expression of OX40L was significantly higher in the primary tumors of RT plus anti-PD-1 or plus HHP. In abscopal tumors, the expression levels remained largely unchanged.

### 3.4. Hypofractionated Irradiation with 3 × 8 Gy Improves Primary Tumor Control But Fails to Induce Abscopal Effects

**Figure 8:**
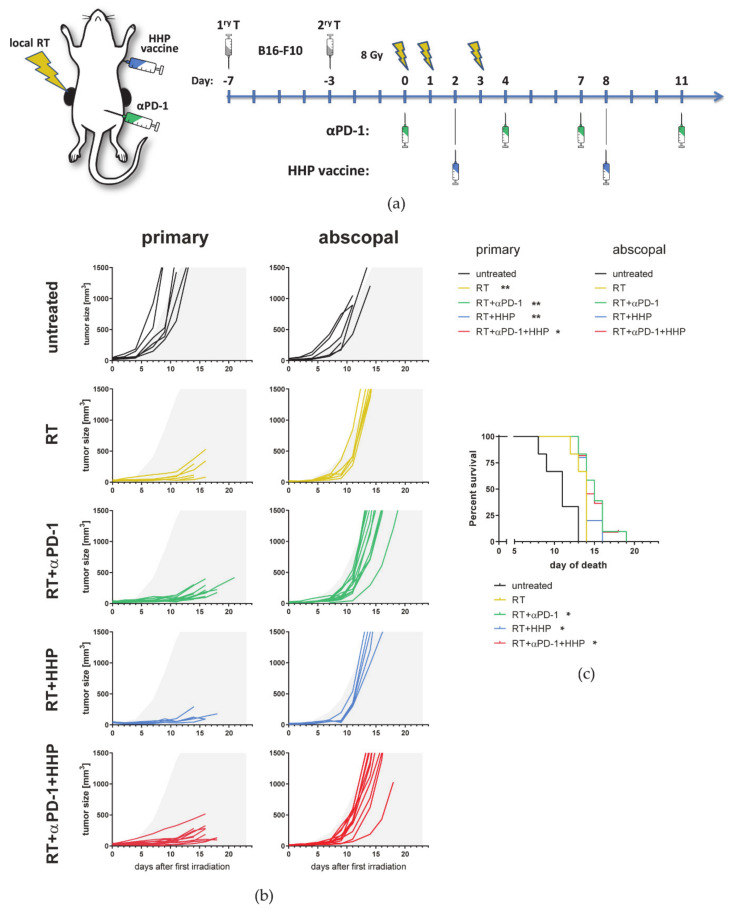
Hypofractionated irradiation with 3 × 8 Gy improves primary tumor control but fails to induce abscopal effects. (**a**) C57Bl/6 mice were subcutaneously injected with 0.2 × 10^6^ B16-F10 tumor cells into the right flank. Four days afterwards, a second tumor was injected on the left flank which later served as the non-irradiated abscopal tumor. The mice received one of the following treatments or combinations thereof. Only the first injected primary tumor was irradiated with 3 × 8 Gy on d0 seven days after injection and on days one and three. Beginning with the first irradiation on d0 the mice were intraperitoneally injected with 200 µg anti-PD-1 antibody (αPD-1) every three to four days for a total of four injections. Additionally, HHP vaccine (5 × 10^6^ cells) was injected twice subcutaneously into the neck on days 2 and 8. (**b**) Individual tumor growth curves are depicted. For a better comparability of the treatment groups, gray areas indicate retarded tumor growth beyond the mean of primary and abscopal tumors of the control group, respectively. A Kruskal–Wallis test with Dunn’s correction for multiple testing was calculated to compare the areas under the individual growth curves of the treatments with untreated controls. (**c**) For the survival a log-rank (Mantel–Cox) test was calculated with Holm–Sidak correction for multiple testing to compare the treatments with the control group. * *p* < 0.05, ** *p* < 0.01; *n* = 5–11 animals per group from two independent experiments.

By increasing the total irradiation dose, we aimed to set a more potent stimulus for the abscopal anti-tumor immune response resulting in an improved treatment outcome. Therefore, we added another fraction of 8 Gy to the treatment schedule on day 1 (Figure 8a).

As expected, 3 × 8 Gy further improved the local tumor control of the primary tumor. Therefore, immunotherapies had no additional effect on the growth of the primary tumor (Figure 8b). Most strikingly, the abscopal tumor growth was not markedly affected by any treatment, even not by combination of RT plus anti-PD.1. The survival of the mice was significantly prolonged in all radioimmunotherapy groups compared to untreated control mice (Figure 8c).

### 3.5. The Number of 8 Gy Fractions Varies Cytokine and Immune Checkpoint Ligand Expressions

**Figure 9:**
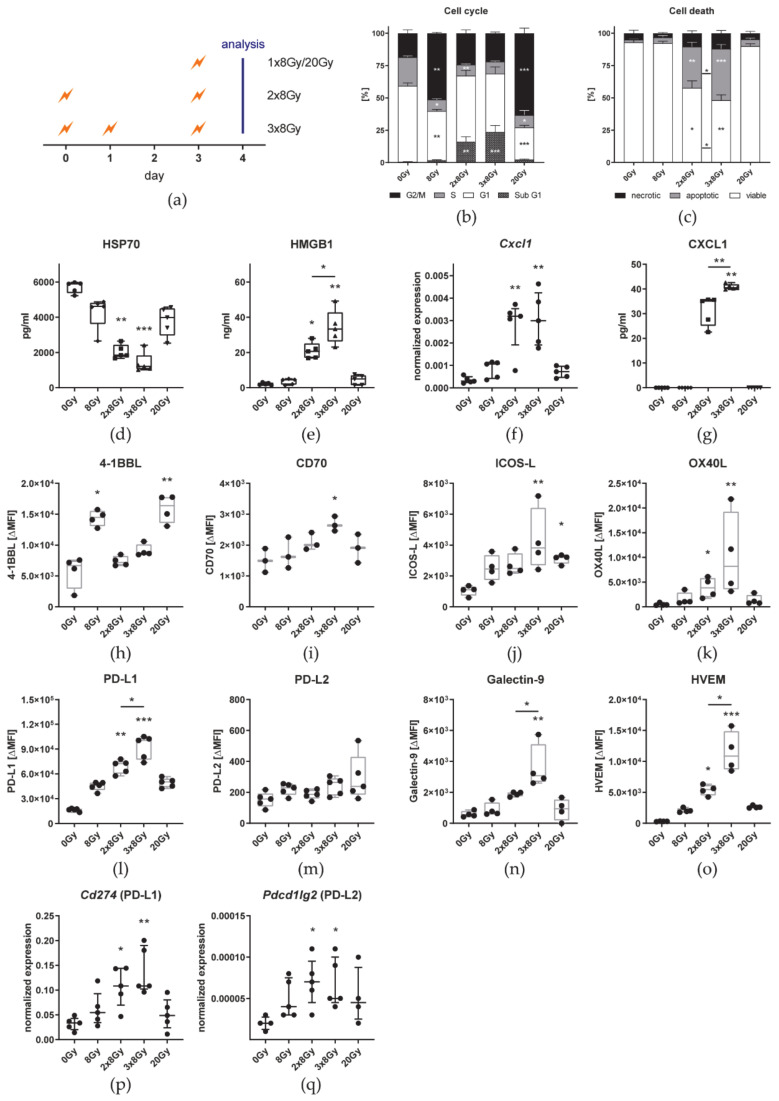
Hypofractionated irradiation with 3 × 8 Gy significantly increases HMGB1 release and PD-L1, Galectin-9, and HVEM expression on tumor cells compared to 2 × 8 Gy. (**a**) B16-F10 cells were seeded 16 h before d0. The 2 × 8 Gy treatment group was irradiated on d0 and d3. One additional fraction of 8 Gy was applied on d1 in the 3 × 8 Gy group. Single fraction groups were irradiated with 8 Gy or 20 Gy on d3. Twenty-four hours after the last irradiation, cell-free supernatants were harvested for ELISA and the cells for flow cytometry and qPCR. The cell cycle (**b**) and cell death (**c**) analyses are shown as stacked bar charts showing the mean ± SD. The concentration of the two DAMPs HSP70 (**d**) and HMGB1 (**e**), and CXCl1 (**g**) in the cell culture medium is depicted as box plots with whiskers from minimum to maximum values. The expression of *Cxcl1* (**f**), *Cd274* (**p**), and *Pdcd1lg* (**q**) is displayed as normalized expression (median ± interquartile range) to the three housekeeping genes *Hprt*, *Tbp*, and *Rps18*. H-O: The autofluorescence corrected ΔMFI (median fluorescence intensity) of 4-1BBL (**h**), CD70 (**i**), ICOS-L (**j**), and OX40L (**k**), PD-L1 (**l**), PD-L2 (**m**), Galectin-9 (**n**), and HVEM (**o**) is shown as box plots with whiskers from minimum to maximum values. A Kruskal–Wallis test with Dunn’s correction for multiple testing was used to compare the treatment groups with the respective untreated control (0 Gy). Additionally, a Mann–Whitney U test was calculated to compare 2 × 8 Gy and 3 × 8 Gy groups; * p < 0.05, ** *p* < 0.01, *** *p* < 0.001; *n* = 3–5.

As an additional fraction of 8 Gy did not induce significant abscopal immune responses, we aimed to investigate the impact of various fractions of 8 Gy on cell death, DAMPs, immune checkpoint ligands, and cytokine expression in vitro. Therefore, B16 cells were irradiated in the same intervals as in the in vivo setting or with a single fraction of eight or 20 Gy and were analyzed 24 h after the last irradiation (Figure 9a).

Irradiation of B16 cells with a single dose of either 8 Gy or 20 Gy resulted in a significantly larger proportion of cells in the G2/M phase of the cell cycle (Figure 9b) 24 h after the last irradiation. In contrast, fractionated irradiation led to a significantly higher percentage of cells with degraded DNA (sub-G1). However, there was no significant difference between 2 × 8 Gy and 3 × 8 Gy. As detected by AxV/PI staining, only fractionated irradiation induced significant amounts of cell death that was predominantly characterized by apoptosis and was significantly higher after irradiation with 3 × 8 Gy (Figure 9c). Although the release of the danger signal HSP70 into the cell culture medium was lower after any irradiation scheme than from untreated cells, only fractionated irradiation significantly decreased the concentration in the medium (Figure 9d). The other DAMP HMGB1 was detected only in small amounts in the cell culture medium of untreated and 8 Gy or 20 Gy irradiated cells (Figure 9e). B16 cell released significantly more HMGB1 when irradiated with 3 × 8 Gy than with 2 × 8 Gy. In the cell culture medium of non-irradiated and one time irradiated cells, CXCL1 was not detectable, even though low level expression was found in qPCR analysis (Figure 9f,g). After fractionated irradiation mRNA expression significantly increased and protein release was detectable by ELISA. Furthermore, CXCL1 was significantly more abundant in the medium of 3 × 8 Gy than 2 × 8 Gy irradiated B16 cells.

The surface expression of immune checkpoint ligands after irradiation was analyzed by flow cytometry and qPCR analysis. Even though untreated B16 already cells express PD-L1 on the cell surface, it is further up-regulated by irradiated cells in general (Figure 9l). Specifically, fractionated irradiation with 3 × 8 Gy further increases PD-L1 expression significantly more than 2 × 8 Gy. The same pattern could be confirmed by qPCR analysis (Figure 9p). Although *Pdcd1lg2* (PD-L2) was significantly higher expressed on mRNA level after fractionated irradiation, no changes were found for the surface expression (Figure 9m,q). Furthermore, expression levels detected by both methods were very low. Similar to PD-L1, the highest surface expression of the other two inhibitory immune checkpoint ligands Galectin-9 (Figure 9n) and HVEM (Figure 9o) and the stimulatory counterparts CD70 (Figure 9i), ICOS-L (Figure 9j), and OX40-L (Figure 9k) was found in B16 cells irradiated with 3 × 8 Gy. Unlike the other checkpoint ligands, 4-1BBL was significantly higher expressed on single dose irradiated cells than on untreated cells and after fractionated irradiation (Figure 9h).

**Figure 10:**
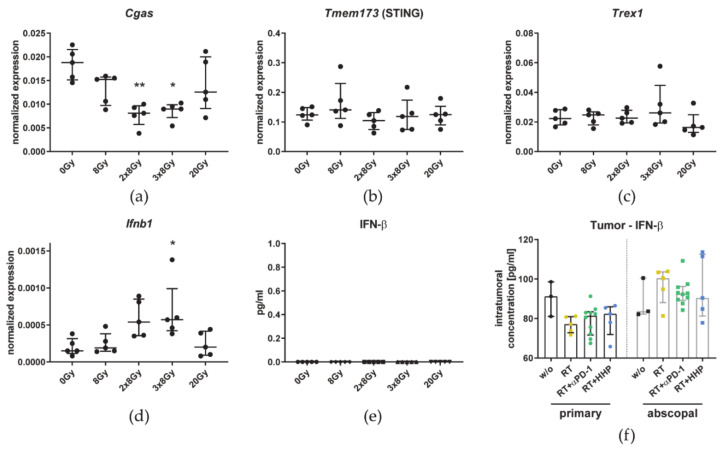
Tumor irradiation with 3 × 8 Gy has no significant impact on the cGAS/STING-related IFN-β pathway. B16-F10 cells were seeded 16 h before d0. The 2 × 8 Gy treatment group was irradiated on d0 and d3. One additional fraction of 8 Gy was applied on d1 in the 3 × 8 Gy group. Single fraction groups were irradiated with 8 Gy or 20 Gy on d3. Twenty-four hours after the last irradiation, cell-free supernatants were harvested for ELISA and the cells qPCR. A-D: The expression of *Cgas* (**a**), *Tmem173* (**b**), *Trex1* (**c**), and *Ifnb1* (**d**) is displayed as normalized expression (median ± interquartile range) to the three housekeeping genes *Hprt*, *Tbp*, and *Rps18*. The concentration of IFN-β in the cell culture medium (**e**) and in tumor lysates (RT = 2 × 8 Gy) (**f**) is depicted as bar graphs with individual values and interquartile range. A Kruskal–Wallis test with Dunn’s correction for multiple testing was used to compare the treatment groups with the respective untreated control (0 Gy). Additionally, a Mann–Whitney U test was calculated to compare 2 × 8 Gy and 3 × 8 Gy groups; * *p* < 0.05, ** *p* < 0.01; *n* = 5 in vitro, *n* = 3–10 animals per group in vivo.

Additionally, we analyzed the impact of the fractionation on the cGAS/STING pathway. The expression of *Cgas* was reduced after fractionated irradiation (Figure 10a). *Tmem173* (STING) and *Trex1* expression was not affected by irradiation (Figure 10b,c). While *Ifnb1* expression on mRNA level was significantly increased in B16 cells irradiated with 3 × 8 Gy, no IFN-β could be detected by ELISA and the cytokine level inside the tumor in vivo was not affected by any treatment (Figure 10d–f).

## 4. Discussion

### 4.1. HHP Vaccines Act Systemically But Only on Previously Irradiated Tumors and Fail to Improve RT + Anti-PD-1 Induced Abscopal Responses

Based on the synergistic effect of RT and HHP vaccination we observed when injecting the vaccine peritumorally [21], we aimed to investigate if HHP vaccines can induce systemic immune responses after injection distant to the tumor. Ohlfest et al. demonstrated in a murine glioma model that the anti-tumor immune response was more effective the further away from the tumor they injected the vaccine. This was dictated by the presence of the tumor itself as the T cell priming efficiency was irrespective of the injection site in tumor-free mice [29]. These limitations can be explained by the immunosuppressive microenvironment of tumor draining lymph nodes mediated by Tregs and cytokines, in which tumor antigens are cross-presentation leads to tolerization [30]. Therefore, we injected the HHP vaccine in the abscopal model distant from the tumors subcutaneously in the neck.

Although injected distantly from the tumor, the HHP vaccine further delayed the tumor growth of the primary irradiated but not the abscopal tumor (Figure 1b). As shown in Figure 9, HMGB1 might be a key player in this scenario, since it is locally released following irradiation with more than one single fraction of 8 Gy. Surprisingly, the addition of the HHP vaccine partly abrogated the abscopal response induced by RT + anti-PD-1 (Figure 1b).

Since hypofractionated protocols are suggested to be more immunogenic compared to norm-fractionated ones [31], we focused on this radiation scheme which is more and more used in the clinics [32]. In preclinical mouse models, the local irradiation with a dose per fraction of 8–10 Gy delivered in two to three fractions has been shown to mimic a hypofractionated irradiation schedule in humans [24,33,34]. Recent research further supports the hypothesis that combined strategies are superior to induce efficient systemic anti-tumor responses [35,36].

Immune phenotyping of primary tumors revealed that the concentration of immune cells was increased after combinations of RT with immunotherapies, especially after RT plus anti-PD-1 (Figure 3b). This combination with the most pronounced abscopal effects was the only treatment in which the concentration of CD45+ cells was also increased significantly in abscopal tumors. These results suggest that first, HHP vaccines act systemically, but only when the tumor microenvironment is modulated by previous irradiation of the tumor. Second, as also observed for many immune cell subtypes (Figure 3 and Figure 4), the concentrations of the triple combination resemble more the RT plus HHP treatment rather than RT plus anti-PD-1, indicating that the vaccination is somehow dominant and that the outcome of combining multiple (immuno)therapies is not necessarily the sum of their individual effects.

### 4.2. RT Plus Anti-PD-1 Induced Abscopal Anti-Tumor Immune Responses Are Associated with an Increased Infiltration of CD8+ T Cells, Monocytes/Macrophages, and Dendritic Cells

Looking at the total number of immune cells in primary and abscopal tumors (Figure 3a) suggests that an increased concentration is not always equivalent to a higher infiltration of those immune cells into the tumor after a certain treatment. While we observed elevated concentrations of leukocytes in primary irradiated tumors and an even significantly higher one when anti-PD-1 was added, total numbers dropped after RT and seemed to even further decrease with vaccination. In a melanoma model, it was found that when a peptide vaccine was injected together with incomplete Freund’s adjuvant to form an antigen depot, the T cells were attracted to the site of vaccination and not to the tumor resulting in impaired tumor control [37]. At the site of HHP vaccine injection, usually a nodule can be seen for several days, probably consisting of residual tumor cells (not shown), indicating that the vaccine might be encapsulated instead of cleared by phagocytes and APCs. Similar to the results of Hailemichael et al., T cells, including most of the subsets and especially NKT cells, were the only cell type of which the concentration in primary tumors was negatively affected by vaccination (Figure 4a,b,d,e). Peritumoral injection of the vaccine might bypass this problem as previously T cells were among those that were significantly more abundant after combined treatment [21]. Although not significant, T cell concentrations in abscopal tumors tended to be lower than those in the untreated group which corroborates the hypothesis that T cells are sequestered by HHP vaccine depots.

In addition to the lower immune cell numbers in irradiated tumors, most of the immune cells decreased in the peripheral blood after RT as well (Figure 6). Leukopenia and impaired functionality of lymphocytes, monocytes, and neutrophils have also been reported during and after RT of patients with cervical, rectal, oral, and breast cancer [38,39,40]. Further, from the conditioning of patients suffering from hematologic malignancies for bone marrow transplantation with total body irradiation, it is known that RT is very immunosuppressive and toxic for immune cells [41]. Therefore, immune cells have long been considered to be upon the most radiosensitive cells in the body and indeed, we have previously shown ex vivo that human immune cells are susceptible even for single doses of ionizing radiation but the radiosensitivity differs between immune cell types. Monocytes appeared to be the most resistant cell type while lymphocytes and NK cells died even after irradiation with doses typically used for the irradiation of tumors. However, even at higher doses, T cells were not fully eradicated, suggesting that the surviving fraction could still exert their tumor killing activity even after irradiation [42]. Arina et al. investigated this issue in a very elegant in vivo setting using a mouse model where they differently labeled T cells in the tumor and in the periphery to discriminate pre-existing T cells from those that newly infiltrated after RT. In accordance with our results, they found that fractionated irradiation or a high single dose decimated pre-existing T cells in the tumor but the majority remained viable and new T cells infiltrated after RT. However, they have shown that these newly tumor-infiltrating T cells contribute to the anti-tumor immune response but pre-existing irradiated T cells can do so as well. Blocking the egress of T cells from lymph nodes with FTY720 just before RT and thereby also the subsequent infiltration into tumors, had no negative impact on the tumor growth. Although the proliferative capacity of the surviving T cells was diminished, they retained their motility and produced even more IFN-γ than non-irradiated T cells [43]. Activated and memory T cells have been shown to be more radioresistant than naïve T cells [44,45] and Arina et al. additionally found TGF-β in the tumor microenvironment to contribute to the resistance of tumor infiltrating T cells [43]. We conclude that although ionizing radiation is harmful for immune cells, the net outcome for the tumor control after RT is positive as it increases the functionality of surviving or newly infiltrating leukocytes and provides the basis for efficient immunotherapies. In contrast to primary tumors, the significantly higher concentration of CD45+ cells in the abscopal tumors of the RT plus anti-PD-1 group was accompanied with a higher total number, indicating immune cell infiltration. These newly infiltrating immune cells largely consisted of monocytes/macrophages, DCs and T cells of which CD8+ T cells were the main subset (Figure 3g,k and Figure 4a,e). Further, it has to be mentioned that the tumor itself can exert effects such as DNA damage in more distant places in the organism. These effects have been proven to be at least partly immune-mediated and macrophage recruitment takes place [46,47]. In line with our findings, the success of immune checkpoint blockade with anti-PD-1 in basal or squamous cell carcinoma patients was mediated by novel T cell clones which had not been present in the same tumors before [48], underlining the different mechanisms in abscopal and primary irradiated tumors.

CD62L is a marker of T cell activation and effector functions. In a melanoma model, Yang et al. demonstrated that CD62L, which is found on naïve T cells, is shed upon T cell activation and is associated with lytic activity [49]. In our model, the percentage of CD4+ and CD8+ T cells negative for CD62L significantly increased in irradiated tumors (Figure 4g,h), indicating increased T cell functionality.

All irradiated primary tumors, and especially those where anti-PD-1 was added, were further enriched for CD11b- DCs (Figure 3h). This subset of DCs can be referred to as conventional type 1 DCs (cDC1s) which are specialized to cross-present antigen to CD8+ T cells and stimulate NK, NKT, and CTLs by producing high amounts of IL-12 [50]. In the tumor microenvironment, cDC1s are associated with the recruitment of effector CD8+ T cells and thus better prognosis for several tumor entities [51]. Further, in pre-clinical trials, cDC1s have been shown to be essential for the treatment with ICIs as the treatment efficacy is abrogated in cDC1 deficient Batf3-/- mice [52,53]. As cDC1s express receptors specific for the recognition of dead and dying cells [54], they might not only be important to initiate CTL responses in irradiated tumors where ICD is induced, but might also be required for the cross-presentation of HHP vaccines and could be targets for further improvements of those vaccines.

TAMs are generally thought to contribute to tumor progression and immunosuppression by various mechanisms such as supporting metastasis, angiogenesis, or T cell inhibition [55]. Some subsets however, are associated with good prognosis. Etzerodt et al. classified three different monocyte and macrophage subsets in the TME based on their Ly6-C and MHC-II expression. These are MNs, iTAMs, and mTAMs which contained a very suppressive CD163+ TAM subset. In a melanoma model, tumor infiltrating MNs were polarized towards immunosuppressive mTAMs. Depletion of these CD163+ cells resulted in increased infiltration of iTAMs which had an inflammatory phenotype and subsequently activated T cells were attracted to promote tumor regression [56]. Similar to CD163+ mTAM depletion, RT and anti-PD-1 significantly shifted the balance towards more iTAMs and less mTAMs in primary tumors (Figure 3m,n).

According to the correlation analysis of immune cell concentrations with the weight of the abscopal tumor in the RT plus anti-PD-1 group (Figure 5), immune cells seem to have differential roles in abscopal responses even though functional tests still need to be carried out in future experiments.

In line with the in vitro results for the immune checkpoint ligands (Figure 9), RT up-regulated the expression of PD-L1, HVEM, and OX40L in primary but not abscopal tumors (Figure 7). Adding either of both immunotherapies further enhanced this effect. Similarly, Dovedi et al. showed that PD-L1 was significantly more abundant on tumor cells of CT26 tumors up to seven days after fractionated RT in combination with anti-PD-L1. In vitro they found that the surface expression was dependent on IFN-γ and could be further increased with TNF-α [57]. Both are cytokines, which we found in higher concentrations in tumor lysates of primary tumors treated with RT or RT plus anti-PD-1 (Figure 4k,m).

In conclusion, tumor growth retardation of locally irradiated primary tumors was accompanied by changes of the immunological microenvironment. The total number of immune cells per tumor was reduced mainly by the elimination of B cells and neutrophils. Although the addition of either HPP vaccines or anti-PD-1 did not change immune cell numbers, primary tumor growth was further reduced. Immunotherapies thus seem to have improved the functionality of immune cells present in the tumors. In contrast, the most prominent difference which we observed between abscopal tumors of treatment combinations that failed to elicit abscopal effects and RT plus anti-PD-1 was the elevated CD8+ T cell infiltration. Other changes in immune cell infiltration, cytokine milieu, and immune checkpoint ligand expression were rather small. This suggests that among all effector cells, including NK cells and the other T cell subsets which we investigated, only CD8+ T cells mediated abscopal responses and that these are probably newly infiltrating cells rather than pre-existing ones.

### 4.3. Irradiation with 3 × 8 Gy Fails to Induce Prominent Abscopal Effects by Increasing the Expression of Suppressive Immune Checkpoint Molecules

Albeit the local tumor control of primary irradiated tumors was improved by adding another fraction of 8 Gy, the abscopal anti-tumor immune responses we observed when we treated mice with 2 × 8 Gy and anti-PD-1 (Figure 1) were almost completely abrogated using 3 × 8 Gy (Figure 8). The survival benefit, which was still significant in the radioimmunotherpy groups, was therefore only due to the very good local tumor control of the irradiated tumor.

To investigate the underlying mechanism on the tumor cell level, we used the same irradiation schedules in vitro compared to a single irradiation with 8 Gy or a single high dose (20 Gy). Cell death in 3 × 8 Gy irradiated B16 cells was higher than with 2 × 8 Gy which was characterized by a significantly increased rate of apoptosis (Figure 9b,c). The phagocytosis of apoptotic cells was shown to polarize macrophages towards an immunosuppressive phenotype [58] and thus contributes to tumor progression [59]. As one of the best known DAMPs and characteristics of ICD, HMGB1 is a potent stimulus for immune responses [60]. The release of HMGB1 by irradiated tumor cells, that we have detected especially with 3 × 8 Gy (Figure 9e), was however shown to promote proliferation of living tumor cells [61]. CXCL1, a chemokine originally identified in melanoma, is associated with oncogenesis, tumor progression, and metastasis across several tumor entities [62] by recruiting tumor-associated neutrophils [63]. Moreover, *Cxcl1* expression was reported to be higher in immunotherapy-resistant tumor cell clones [64]. *Cxcl1* expression was low in B16 cells but increased upon fractionated irradiation (Figure 9f) and only there CXCL1 could be detected in the cell culture supernatant in significant higher amounts with 3 × 8 Gy (Figure 9g). In the serum of patients with colorectal cancer, CXCL1 is a predictive marker for lung and liver metastases [65]. Albeit, ionizing radiation, administered either fractionated or as a single dose, increased the surface expression of most of the immune checkpoint ligands on B16 cells, it were predominantly the immunosuppressive ones (PD-L1, Galectin-9, and HVEM) that were up-regulated. Further, the very same were significantly higher after 3 × 8 Gy compared to 2 × 8 Gy whereas no differences between both fractionations were found for the stimulatory ligands 4-1BBL, CD70, ICOS-L, and OX40L (Figure 9h–o), in sum indicating a more suppressive tumor cell phenotype at 3 × 8 Gy. Vanpouille-Box et al. reported that upon irradiation (e.g., 3 × 8 Gy) DNA is released into the cytosol of the cell which is sensed by the cGAS/STING pathway and ultimately leads to the secretion of the inflammatory cytokine IFN-β. If the dose is too high (e.g., 20 Gy), however, the exonuclease Trex1 is expressed, degrades cytosolic DNA, and thereby shuts down the type I IFN response [27]. While *Ifnb1* expression on mRNA level was significantly increased in B16 cells irradiated with 3 × 8 Gy, no IFN-β could be detected in the cell culture supernatant at any condition and the cytokine level inside the tumor in vivo was not affected by any treatment (Figure 10), suggesting that this pathway does not play a role in B16 melanoma.

Although most of the differences between irradiation with 2 × 8 Gy and 3 × 8 Gy in vitro and in vivo were significant, the absolute changes were rather little. It might be the sum of these small changes that contributes to the failure of 3 × 8 Gy to elicit abscopal effects. However, the mechanistic basis of these associations needs further evaluations. In the work by Zhang et al., a comparable irradiation scheme with 3 × 9.18 Gy plus anti-PD-1 checkpoint inhibition was able to induce abscopal anti-tumor immune responses in the B16-CD133 model. When they used the B16 wildtype model without exogenous antigen however, similar to ours results, no abscopal effects were observed [66].

## 5. Conclusions

Altogether, our results suggest that the immunotherapies profit from the RT-induced TME modulations, but instead of pure infiltration of immune cells in general or specific subsets, it is rather a functional alteration of pre-existing or newly infiltrating immune cells that mediate the improved anti-tumor immune response. These mechanisms need to be addressed in future experiments. HHP vaccines had systemic effects only on previously irradiated tumors and vaccination partly abrogated abscopal effects induced by RT and anti-PD-1 checkpoint inhibition, indicating that both immunotherapies act through different mechanisms which negatively influence each other. These limitations might be overcome in the future by improving HHP vaccines with adjuvants such as poly(I:C) [67] or anti-CTLA-4 to aid in the priming of CTLs [68].

Conventional type 1 DCs, which we found to be more abundant in irradiated tumors, are becoming increasingly recognized for their importance to prime and activate CD8+ T cells by the uptake and cross-presentation of tumor antigens [54]. Direct targeting of those cells or relocating the injection point to a peritumoral injection of the vaccine after irradiation, a point where cDC1s are already present, might additionally improve HHP vaccine efficiency.

## Data Availability

The data presented in this study are available on reasonable request from the corresponding author.

## Supplementary Materials

The following are available online at https://www.mdpi.com/2072-6694/13/4/714/s1, Figure S1: Gating strategy of the immune phenotyping panel 1, Figure S2: Gating strategy of the immune phenotyping panel 2, Figure S3: Gating strategy of the immune phenotyping panel 3, Table S1: Antibodies and dyes of immune phenotyping panel 1, Table S2: Antibodies and dyes of immune phenotyping panel 2, Table S3: Antibodies and dyes of immune phenotyping panel 3, Table S4: Antibodies and dyes of immune checkpoint panel 1, Table S5: Antibodies and dyes of immune checkpoint panel 2, Table S6: List of Bio-Rad Prime PCR Primers.
