# Supplementary material for: Combinations of Radiotherapy with Vaccination and Immune Checkpoint Inhibition Differently Affect Primary and Abscopal Tumor Growth and the Tumor Microenvironment
Source: Cancers (Basel). 2021 Feb 9;13(4):714. doi: 10.3390/cancers13040714 (PMC7916259; doi:10.3390/cancers13040714)
Supplement: Supplementary file 1 [file cancers-13-00714-s001.pdf]

Supplementary Materials:

Combinations of Radiotherapy with Vaccination and Immune Checkpoint Inhibition Differently Affect Primary and Abscopal Tumor Growth and the Tumor Microenvironment

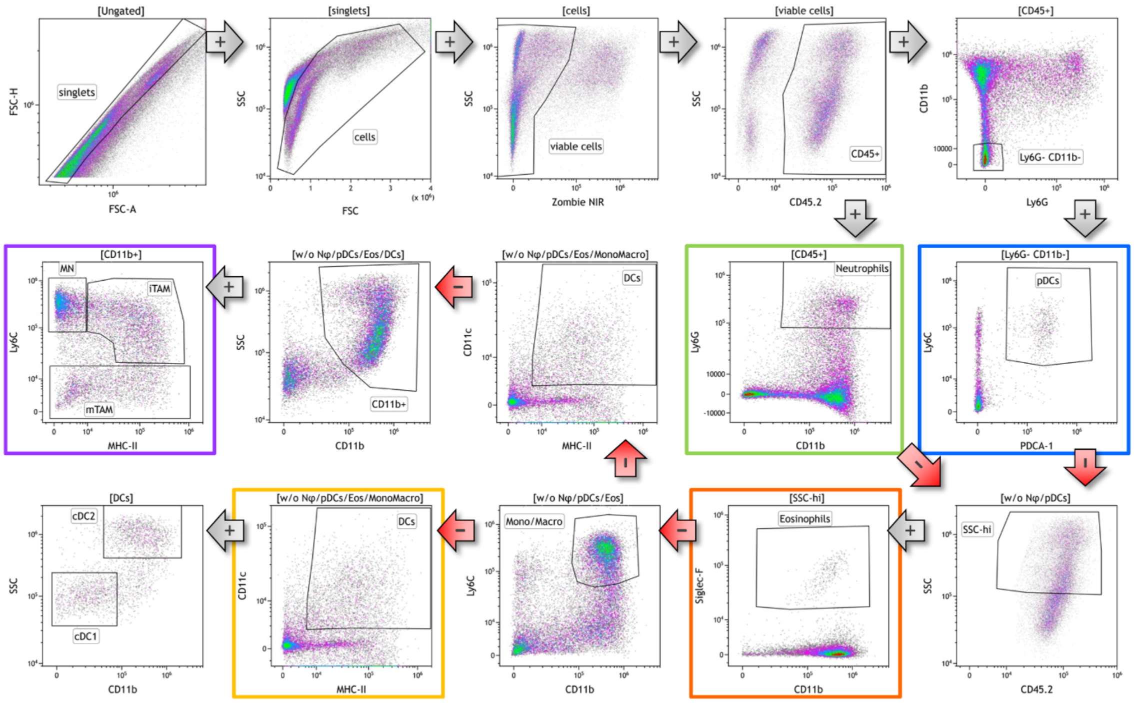

Figure S1. Gating strategy of the immune phenotyping panel 1.After pre-gating on single (FSC-A/FSC-H) viable cells (FSC/SSC, Zombie^{-}) all immune cells were gated as CD45^{+}. Ly6G^{-}, CD11b^{-} plasmacytoid dendritic cells (pDCs) were identified as Ly6C^{+}, PDCA-1^{+}. Neutrophils were gated as CD11b^{+}, Ly6G^{+}, Eosinophils as SSC^{hi}, CD11b^{+}, Siglec-F^{+}, DCs as MHC-II^{+}, CD11c^{+} and subdivided in conventional type 1 DCs (cDC1s; CD11b^{-}, SSC^{lo}) and cDC2s (CD11b^{+}, SSC^{hi}). After pre-gating on CD11b^{+} cells Monocytes (MN) were identified as MHC-II^{-}, Ly6C^{+}, immature tumor associated macrophages (iTAM) as MHC-II^{+}, Ly6C^{+} and mature TAMs (mTAM) as Ly6C^{-}. Major subtypes are highlighted with colored frames. Grey arrows with a "+" indicate subgating of the previous population. The exclusion of the previously gated population from further analysis by Boolean gating is indicated by red arrows with a "-".

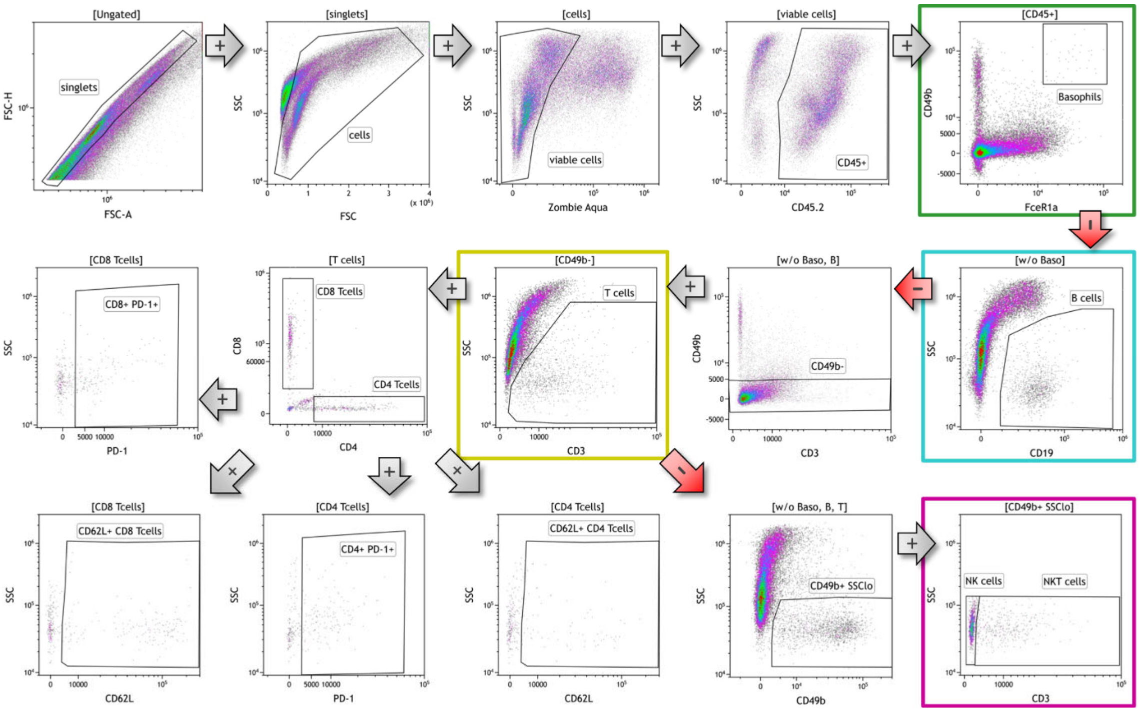

Figure S2. Gating strategy of the immune phenotyping panel 2.After pre-gating on single (FSC-A/FSC-H) viable cells (FSC/SSC, Zombie^{-}) all immune cells were gated as CD45^{+}. Basophils were identified as FcεR1α^{+} CD49b^{+}, B cells as CD19^{+}, T cells as CD49b^{-} CD3^{+}, NK cells as CD49b^{+} SSC^{lo} CD3^{-} and NKT cells as CD49b^{+} SSC^{lo} CD3^{+}. T cells were further subdivided in CD4^{+} and CD8^{+} T cells and analyzed for PD-1 and CD62L expression. Major subtypes are highlighted with colored frames. Grey arrows with a “+” indicate subgating of the previous population. The exclusion of the previously gated population from further analysis by Boolean gating is indicated by red arrows with a “-”.

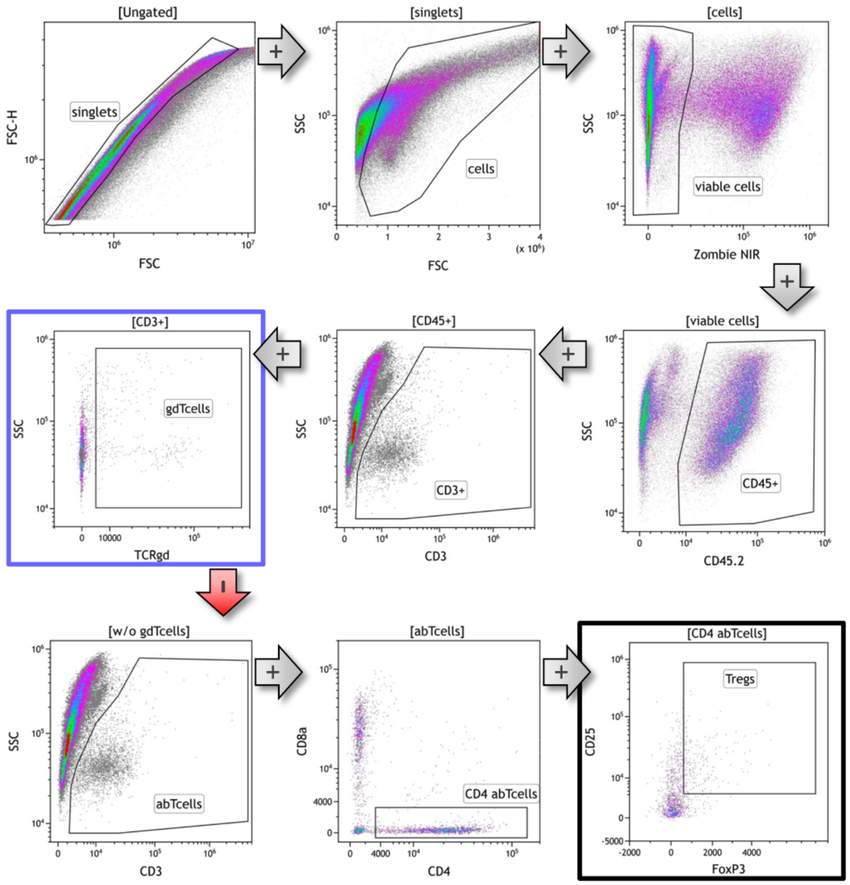

Figure S3. Gating strategy of the immune phenotyping panel 3.After pre-gating on single (FSC-A/FSC-H) viable cells (FSC/SSC, Zombie$^-$) all immune cells were gated as CD45$^+$. γδ T cells were identified as CD3$^+$ TCRγδ$^+$ and Tregs as CD3$^+$ CD4$^+$ CD25$^+$ FoxP3$^+$. Major subtypes are highlighted with colored frames. Grey arrows with a "+" indicate subgating of the previous population. The exclusion of the previously gated population from further analysis by Boolean gating is indicated by red arrows with a "-".**Antibody panels for the immune phenotyping of immune cells in tumors and blood**

Table S1. Antibodies and dyes of immune phenotyping panel 1.

Target	Fluorochrome	Supplier	Catalog #
Live/dead	Zombie NIR	BioLegend	mic
MHC-II (I-A/I-E)	eFluor 450	eBioscience	48-5321
Ly-6G	PE-Cy7	BD	560601
Ly-6C	FITC	BD	553104
CD11b	APC	eBioscience	17-0112
Siglec-F	PE	BD	552126
CD45.2	PerCP-Cy5.5	eBioscience	45-0454
PDCA-1	BV 650	BioLegend	127019
CD11c	BV 510	BD	562949

Table S2. Antibodies and dyes of immune phenotyping panel 2.

Target	Fluorochrome	Supplier	Catalog #
CD62L	PE-Cy7	BD	560516
CD8a	BV 605	BioLegend	100744

FcεR1α	PE	BioLegend	134308
Live/dead	Zombie Aqua	BioLegend	423102
CD4	FITC	BD	553729
CD19	APC-Cy7	BD	561737
CD3e	V450	BD	560801
CD45.2	PerCP-Cy5.5	eBioscience	45-0454
CD49b	APC	BioLegend	108910
PD-1	PE-Dazzle 594	BioLegend	109116

Table S3. Antibodies and dyes of immune phenotyping panel 3.

Target	Fluorochrome	Supplier	Catalog #
Live/dead	Zombie NIR	BioLegend	423106
CD8a	BV 605	BioLegend	100744
CD4	FITC	BD	553729
CD3e	V450	BD	560801
γδTCR	PE	eBioscience	12-5711-82
CD45.2	PerCP-Cy5.5	eBioscience	45-0454
CD25	PE-Cy7	BD	552880
FoxP3	APC	Miltenyi Biotec	130-093-013

Antibody panels for the immune checkpoint ligands

Table S4. Antibodies and dyes of immune checkpoint panel 1.

Table.	Fluorochrome	Supplier	Catalog #
PD-L1	PE-Cy7	eBioscience	25-5982
PD-L2	APC	BioLegend	107210
OX40L	PerCP-eFluor710	eBioscience	46-5905-80
ICOS-L	PE	BioLegend	107405
Live/dead	Zombie Aqua	BioLegend	423102

Table S5. Antibodies and dyes of immune checkpoint panel 2.

Target	Fluorochrome	Supplier	Catalog #
4-1BBL	VioBright-FITC	Miltenyi Biotec	130-105-857
Galectin-9	BV421	BD	566028
HVEM	APC	BioLegend	136305
CD70	PE	eBioscience	12-0701
Live/dead	Zombie Yellow	BioLegend	423104

Quantitative real-time PCR (qPCR)

Table S6. List of Bio-Rad Prime PCR Primers.

Gene symbol	Gene name	Assay ID
Cd274	CD274 antigen (PD-L1)	qMmuCID0011907
Cd70	CD70 antigen	qMmuCED0045283
Cxcl1	chemokine (C-X-C motif) ligand 1	qMmuCED0047655
E330016A19Rik	RIKEN cDNA E330016A19 gene (cGAS)	qMmuCID0025813
Hprt	hypoxanthine guanine phosphoribosyl transferase	qMmuCED0045738
Icosl	Icos ligand	qMmuCID0019198
Ifnb1	interferon beta 1	qMmuCED0050444
Pdcd1lg2	programmed cell death 1 ligand 2 (PD-L2)	qMmuCID0011922

Rps18	ribosomal protein S18	qMmuCED0045430
Tbp	TATA box binding protein	qMmuCID0040542
Tmem173	transmembrane protein 173 (STING)	qMmuCED0046127
Tnfrsf14	tumor necrosis factor receptor superfamily, member 14 (herpesvirus entry mediator; HVEM)	qMmuCID0010946
Tnfsf4	Tumor necrosis factor (ligand) superfamily, member 4 (OX40L)	qMmuCID0005677
Tnfsf9	tumor necrosis factor (ligand) superfamily, member 9 (4-1BBL)	qMmuCED0004214
Trex1	three prime repair exonuclease 1	qMmuCED0061616
